# Antioxidant effect of genistein on ovarian tissue morphology, oxidant and antioxidant activity in rats with induced polycystic ovary syndrome

**DOI:** 10.18502/ijrm.v17i1.3816

**Published:** 2019-03-07

**Authors:** Samira Rajaei, Alireza Alihemmati Ph.D., Ali Abedelahi Ph.D.

**Affiliations:** ^1^Stem Cell Research Center, Tabriz University of Medical Sciences, Tabriz, Iran.; ^2^Department of Anatomical Sciences, Tabriz University of Medical Sciences, Tabriz, Iran.

**Keywords:** *Genistein*, * Antioxidant*, * Ovary*, * Polycystic*, * Follicle*, * Rats.*

## Abstract

**Background:**

Oxidative stress is the most frequent cause of female infertility disorders including polycystic ovary syndrome (PCOS). Genistein as a major component of soybean isoflavone scavenges free radicals by antioxidant activities.

**Objective:**

The present study examines the antioxidant effects of genistein on ovarian tissue following experimental PCOS in rats.

**Materials and Methods:**

Twenty female Wistar rat were randomly divided into the following groups (*n*=5 each group): (I) control group (no treatment); (II) induced PCOS (injection of estradiol valerate); (III) genistein-treated non-PCOS (received genistein); and (IV) genistein-treated PCOS groups. The weight of rats were measured and the blood samples collected and centrifuged. The oxidant and antioxidant activity of plasma and ovaries were measured. All rats were sacrificed under anesthesia, and ovaries were collected and weighted. Histological examination and follicular quality were assessed by staining.

**Results:**

In histological observation, the induced PCOS rats displayed more number of atretic follicles and the follicular quality in genistein-treated rats was similar to the control groups. The plasma and ovaries malondialdehyde levels significantly increased in PCOS rats (p < 0.001), while the total antioxidant capacity levels, glutathione peroxidase, and superoxide dismutase activities significantly decreased (p < 0.001). The plasma and ovary malondialdehyde levels significantly decreased in PCOS rats that were treated with genistein (p < 0.001) and the total antioxidant capacity (p < 0.05), glutathione peroxidase, and superoxide dismutase activities significantly increased (p < 0.001).

**Conclusion:**

Treatment with genistein preserved follicular quality by increasing antioxidant activities and scavenging oxidant levels in PCOS rats.

## 1. Introduction

Polycystic ovary syndrome (PCOS) is the most common endocrine disorder that characterized by hyperandrogenism, an ovulation, menstrual irregularity and metabolic syndrome (1, 2). However, several genetic and environmental factors interface in PCOS condition (3).

Rezvanfar and colleagues demonstrated that oxidative and nitrosative stress have critical role in the pathogenesis of PCOS (4). In normal condition, the low concentrations of reactive oxygen species (ROS) are essential for the physiological functions of female reproductive tract including oocyte maturation, ovulation, fertilization, and implantation (5). High concentration of the ROS or oxidative stress is a common pathological process involved in the process of cell injury (6). It is seen in the elementary phases of most PCOS patients, which disrupts the follicular development in the ovaries and leads to the development of PCOS.

Although all organisms need to make a balance between ROS production and antioxidant system including superoxide dismutase (SOD), catalase (CAT), and glutathione peroxidase (GPx). In the overproduction of ROS such as PCOS conditions, these protective systems are insufficient to entirely scavenge of free radicals and prevent follicular damages.

Zhang and colleagues' studies also support the hypothesis that the oxidant levels were increased in PCOS patients, whereas the antioxidants level was decreased (7).

Currently, studies are still being conducted to find better natural antioxidants for improving all the clinical features of PCOS. Therefore, antioxidant supplementation may be a goal strategy for restricting oxidative stress damages in PCOS patients and thereby improving the quality of ovarian follicles and restoration of reproductive cycle (8).

Genistein is the predominant isoflavones phytoestrogen in soybeans and is a major source of xenoestrogen exposure in both humans and animals (9). Recently, it has been shown that genistein has potential anticarcinogenic activity and could diminish menopause-associated cognitive deficits through antioxidant function and scavenging free radicals (9, 10). Additionally, genistein exhibits both proliferative (estrogenic) and antiproliferative (antiestrogenic) properties (11), and currently dietary supplements of genistein are widely used for menopausal symptoms by women as a potential alternative therapy (12). Previous studies revealed that genistein could activate antioxidant defense systems/pathways which would diminish the oxidative stress activity (13, 14).

The potential mechanisms for the antioxidant effects of genistein on induced PCOS are poorly understood. The effects of genistein on ovarian tissue structure still need further investigation.

We hypothesized that low dose of genistein treatment would exert its antioxidative effect in ovarian follicles, which may partially alleviate the toxic effects induced by PCOS.

In this research study, we tried to investigate the beneficial effect of genistein against induced PCOS as well as the number and quality of ovarian follicles. We also investigated the body and ovary weight of PCOS rats in response to the treatment with genistein.

## 2. Materials and Methods

### Chemicals

All media and chemicals were purchased from Sigma-Aldrich unless otherwise mentioned.

### Animals and experimental design

**Figure 1:**
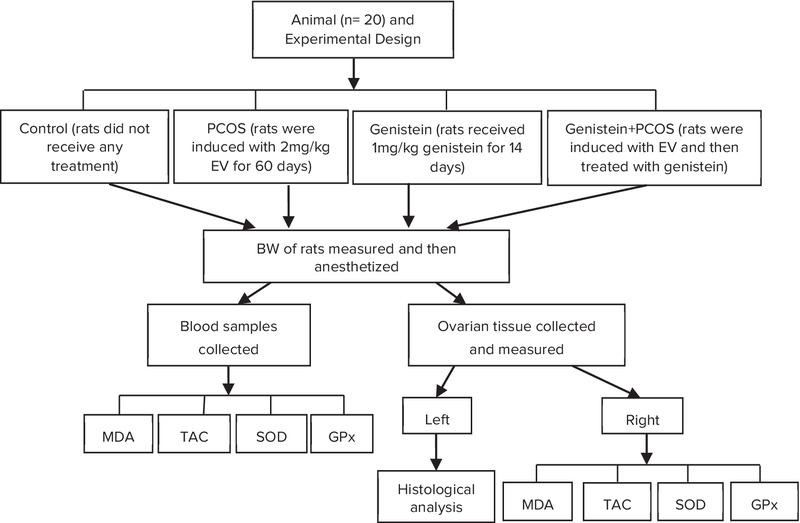
Flow chart of the experimental design. PCOS: polycystic ovary syndrome; MDA: malondialdehyde; TAC: total antioxidant capacity; SOD: superoxide dismutase; GPx: glutathione peroxidase.

Twenty female Wistar albino rats (8-weeks old) with an average weight of 200±20 gram were provided by the International Animal Care and Use Committee at Tabriz University of Medical Sciences. The mice were housed with a controlled cycle (12-hr light/12-h dark at a temperature of 22–24ºC) and had free access to food and water. The flow chart of the study is shown in Figure 1.

The animals were randomly divided into four groups (*n* = 5/each) as follows:

• Group (I) rats were not injected and did not receive any treatment (control group).
• Group (II) PCOS rats were induced by EV for 60 days (induced PCOS group).
• Group (III) rats received genistein for 14 days (genistein-treated non-PCOS group).
• Group (IV) PCOS rats were induced by EV (60 days) and then treated with genistein for 14 days (genistein-treated PCOS group).

## 3. Induction PCOS model and determining the sexual cycle

The injection site was sterilized and 2mg/kg body weight (BW) single dose of EV (Aburaihan, Iran) was injected subcutaneously for 60 days. At the end of the treatment, the body and ovary weights of PCOS rats were measured. The induction of PCOS was verified by vaginal smears and was examined histologically and serologically for a period of 60 days. In this procedure, estrous cycles were monitored under a light microscope for the relative abundance of leukocytes, epithelial, and cornified cells.

### Preparation and administration of genistein

Genistein was obtained from Santa Cruz Company in America. About 0.2 mg of genistein was dissolved in Dimethyl sulfoxide under standard conditions (away from sunlight, moisture, microbial contamination) and then stored at a temperature of –20ºC. The equivalent dietary intakes of phytoestrogens/BW was estimated in humans and rodents by this theory:

The PCOS/non-PCOS rats received 1 mg/kg BW genistein subcutaneously for 14 days at 10 am because of an average 60–70 mg adult would consume phytoestrogen approximately 60–75 mg per day (13, 15).

After the last treatment, the BW of rats were measured and then anesthetized, and 5 mL blood samples were directly taken from the heart. Blood samples were centrifuged at 3000 ×g for 10 min and the separated plasma stored at –70ºC until assayed for oxidant marker (MDA) and antioxidant markers (TAC, SOD, and GPx). All rats (*n*=20) were sacrificed under anesthesia, their ovaries were collected and measured. The left ovaries were immediately fixed in 4% (w/v) paraformaldehyde for histological analysis and the right ovaries were immediately frozen at –70ºC for determining oxidant-antioxidant markers (MDA, TAC, SOD, and GPx level).

### Histological analysis 

The collected ovaries were fixed in 10% buffer formalin, dehydrated by using ethanol, clarified with xylene, embedded by using paraffin wax, and prepared serially sectioned (5 μm). The slides were stained with hematoxylins and eosin (H&E) and visualized under a light microscope with 400 magnification.

Ovarian follicles were categorized according to granulosa cells and layers around the oocyte:

• Primordial follicles (one layer of flattened granulosa cells)
• Primary follicles (one layer of cuboidal granulosa cells)
• Preantral follicles (two or more layers of cuboidal granulosa cells, no antral cavity),
• Antral follicles (numerous layers of cubical granulose cells with antral cavity)

All follicles were numbered when the nucleus of the oocyte was observed (13, 16). Follicular quality was evaluated as normal (if they had an intact oocyte and surrounded by a complete layer of granulosa cells) and atretic (if they were composed of vacuolization, pyknotic nuclei in granulosa cells and contained an occasional shrinkage of oocyte)

### Measurement the LPO level in plasma and ovarian tissue 

Free radical (lipid peroxidation) levels were measured in plasma and ovarian tissue samples using a commercial kit (ZellBio GmbH Kit, Germany) by measuring MDA levels based on thiobarbituric acid reacting substance method. The LPO level was detected spectrophotometrically at 535 nm and evaluated in μmol/mg protein.

### Measurement of the total antioxidant capacity, SOD, and GPx activities in plasma and ovarian tissue 

The TAC, GPx, and SOD activities in plasma and ovaries of control and experimental groups was measured spectrophotometric using a commercial kit (ZellBio GmbH Kit, Germany). The TAC (mmol/L) assay was based on reducing the tripiridyltriazine (TPTZ)–Fe3+ complex to TPTZ–Fe2+ complex with a blue color and maximum absorbance at 593. The GPx activity (units/mg protein) was determined based on coupling the oxidation of glutathione and nicotine adenosine dinucleotide phosphate using glutathione reductase. The SOD activity (units/mg protein) was assessed colorimetrically at 420 nm by the conversion of superoxide anion to hydrogen peroxide and oxygen.

### Ethical consideration

All experiments were carried out in compliance with the guideline developed by the Medical Ethics Committee of Tabriz University of Medical Sciences (1395.249).

### Statistical analysis 

Data were analyzed using statistical SPSS software (Statistical Package for the Social Sciences, version 16.0, SPSS Inc, Chicago, Illinois, USA). The distribution of data was evaluated by Anderson darling test. All data were normally distributed. The variables with normal distribution results were expressed as mean±SD. The oxidant levels and antioxidant activities were evaluated by One-way ANOVA followed by post-hoc analysis Tukey's multiple comparison test. Body and ovarian weight analysis was evaluated by two-way ANOVA, followed by Bonferroni post-hoc test; p < 0.05 was considered as significant.

## 4. Results

### Effect of genistein treatment on body and ovary weight in the induced PCOS rats

**Figure 2:**
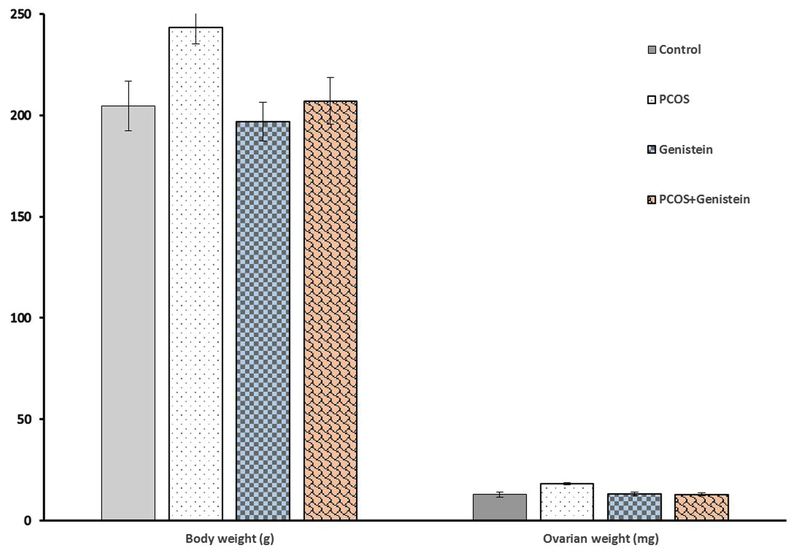
Effect of genistein treatment on BW (g) and ovary weight (mg) in induced PCOS rats. The BW and ovary weights significantly increased in PCOS rats in comparison to the other rats and treatment with genistein significantly decreased the BW and ovary weight. All data were shown as mean±SD, p < 0.05 was considered as significant. ${}^{*}$Represents a significant difference between PCOS and other groups (p < 0.001); PCOS: polycystic ovary syndrome.

BW and ovary weight of rats in the control and experimental groups are shown in Figure 2. The BW and ovary weight increased significantly in PCOS rats in comparison to the control rats (p < 0.001). Treatment with genistein significantly decreased the BW and ovary weight in PCOS rats after 14 days (p < 0.001). The BW and ovary weight were not significant between the genistein-treated groups and the control groups.

### Histological analysis of ovarian tissue

**Figure 3:**
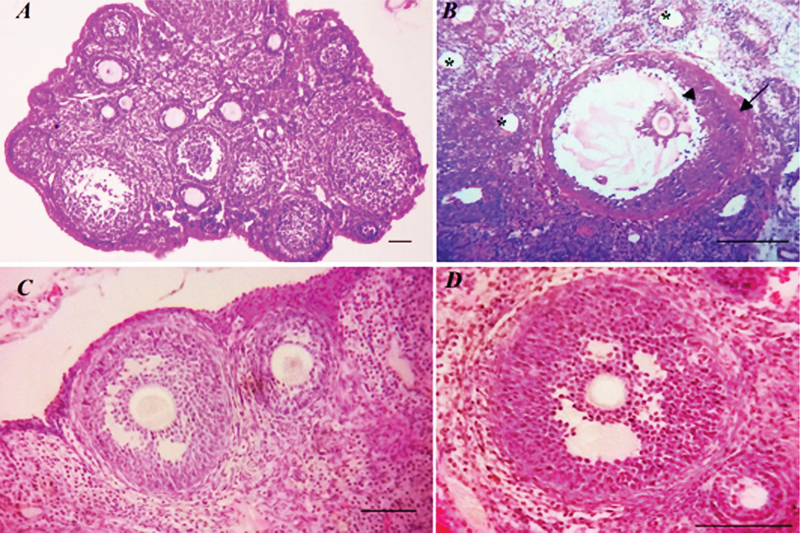
Histological changes in ovarian tissue. (A) Control group, showed normal ovarian morphology. (B) PCOS group, displayed a considerable increase in the number of atretic follicles (star), disruption between granulosa cell layer and oocyte (arrow head) and hyperplasia of theca layer (black arrow). (C) Genistein group, no changes were observed in the histological structure of ovaries. (D) Treated PCOS rats with genistein, observed normal morphology ovaries with a few atretic follicles. Scale bar=100 µm.

Histological structure of normal and atretic follicles was analyzed by light microscope. In control group, ovarian tissue displayed normal follicles at various stages of development (Figure 3A).

The ovarian tissue of induced PCOS rats displayed more signs of follicular cysts in various sizes. Some antral follicles appeared to be moderately atretic with nuclear pyknosis, fragmentation of granulosa cell layer, disruption of the zona pellucida, and hyperplasia of theca layer (Figure 3B). The ovary of genistein-treated groups was similar to the control groups (Figure 3C).

In contrast to the PCOS group, the ovarian tissue showed well-developed antral follicles in the genistein-treated rats including substantial reductions in total populations of ovarian atretic follicles, normal granulosa cell layer, a defined theca layer, and few corpus lutea (Figure 3D).

### LPO levels in plasma and ovarian tissue 

As shown in Table I, a significantly higher level of MDA were detected in plasma and ovaries (Table I) of PCOS groups when compared to the control groups (p < 0.001). The level of plasma and ovary MDA was significantly lower in genistein-treated PCOs groups in comparison to the induced PCOS groups (p < 0.001). The levels of MDA as a measure of oxidative stress were not statistically significant between genistein-treated PCOS and control groups.

### TAC activity in plasma and ovarian tissue

A significant increase in the level of TAC was found in plasma and ovary of PCOS groups when compared to the control groups (p < 0.05).

TAC levels in plasma and ovary of PCOS significantly increased in the genistein treatment groups in comparison to PCOS groups (p < 0.05). There were no significant changes in the level of TAC in plasma and ovary of genistein-treated groups and control groups.

### SOD and GPx activity in plasma and ovarian tissue

The alterations of SOD and GPx activities in plasma and ovarian tissue of the control groups and experimental groups are shown in Table I. The SOD and GPx activity was significantly decreased in plasma and ovaries of PCOS groups in comparison to the control groups (p < 0.001). Genistein treatment significantly increased the SOD and GPx activity in plasma and ovary in comparison to the induced PCOS rats (p < 0.001). Similar to the SOD activity, GPx activity was not statistically significant between the genistein treatment groups and control groups.

**Table 1:** Effect of genistein treatment on plasma and ovary oxidant marker (MDA) and antioxidant activity (TAC, SOD, GPx) in the induced PCOS rats.


**Group**	**MDA (μmol/mg)**		**TAC (mmol/L)**		**SOD (U/mg)**		**GPx (U/mg)**	
	**Plasma**	**Ovary**	**Plasma**	**Ovary**	**Plasma**	**Ovary**	**Plasma**	**Ovary**
Control	12.09 ± 1.33	21.36 ± 2.66	0.83 ± 0.08	1.59 ± 0.09	2.26 ± 0.23	11.83 ± 1.69	34.56 ± 2.18	120.34 ± 9.96
PCOS	26.05 ± 2.65${}^{**}$	38.38 ± 4.40${}^{**}$	0.57 ± 0.07${}^{*}$	0.59 ± 0.09${}^{*}$	0.98 ± 0.18${}^{**}$	6.04 ± 0.90${}^{**}$	18.97 ± 1.40${}^{**}$	70.82 ± 6.77${}^{**}$
GEN	15.83 ± 2.96	24.43 ± 3.92	0.89 ± 0.06	1.80 ± 0.11	2.66 ± 0.21	14.69 ± 0.81	35.84 ± 2.98	158.67 ± 14.41
PCOS + GEN	14.04 ± 2.16	24.37 ± 2.90	0.90 ± 0.04	1.88 ± 0.09	2.17 ± 0.18	15.18 ± 1.04	36.16 ± 2.38	149.06 ± 10.51
Note: Body and ovary weights of all rats (*n*=20) were measured at the end of treatment. In PCOS rats, 2mg/kg single dose of estradiol valerate was injected subcutaneously for 60 days. Genistein (1 mg/kg) was injected subcutaneously into rats for 14 days. Comparisons among groups were performed using one-way ANOVA. All data were shown as mean±SD. ${}^{*}$Represents a significant difference between PCOS and other groups at p < 0.05 level; ${}^{**}$Represents a significant difference between PCOS and other groups at p < 0.001 level. PCOS: polycystic ovary syndrome; GEN: genistein; MDA: malondialdehyde; TAC: total antioxidant capacity; SOD: superoxide dismutase; GPx: glutathione peroxidase.								

## 5. Discussion

The present study was designed to investigate the effects of single intraperitoneal dose of EV on rat ovaries as a PCOS model and evaluate serum and ovarian tissue oxidant (MDA) and antioxidant (TAC, SOD, GPX) levels in PCOS model. Also, this study is an attempt to examine the effect of genistein on oxidant level, antioxidant activity, and morphology of ovarian tissue in the induced PCOS rats.

The results of this study clearly revealed that 2mg/kg EV injection induced some changes including multisize cysts, irregular estrous cycle, follicular atresia, and increased BW and ovary weight. This result is in accordance with the Walters and colleagues' results that suggested that clinical characteristics of PCOS were induced in rat models by EV (17).

BW and ovary weight increased in PCOS rats during EV supplementation. March and colleagues reported that about 42% of PCOS patients have the complication of obesity (18). The increased body and ovarian weight may be due to the accumulation of fatty tissue, formation of follicular cysts, and accumulation of follicular fluid in the cystic follicles. Our results show that the exposure to genistein significantly decreased BW and ovary weight in PCOS rats that may be responsible for decreasing the fatty formation, decreasing follicular cysts (follicular fluid), and scavenging oxidative stress due to the potential antioxidant capacity. A positive correlations between oxidative stress levels and obesity indexes in the PCOS women were reported in previous studies (19). The present study indicated two types of histologic changes in the ovaries in PCOS rats. The first type was characterized by a high number of atretic and poly-cystic follicles. Such changes are probably associated with the consequence of the accumulation of follicular fluid in the cystic follicles and high levels of estrogens in follicular fluid (20).

The other changes were increasing the thickness of stroma and ovarian capsule, hyperplasia of internal theca cells and anovulation. These changes could be directly related to oxidative stress or indirectly associated with the inhibition of gonadotropins hormones (21). Our study demonstrated more sign of cystic antral follicles during EV injection, suggesting an anovulatory condition in PCOS rats. Pandey and colleagues suggested that high level of androgens in accordance with antioxidants–oxidant imbalance may be responsible for the induction of degenerating follicles, which are the major causes of PCOS induction (22).

Our results demonstrated that genistein-treated ovaries appeared to be healthy with regular granulosa and theca cell layers and well-developed antral follicles. Thus, our histological investigation in accordance with biochemical analysis indicated that genistein can exert beneficial effects in the PCOS condition through decreasing the oxidative stress production or by estrogen pathways. Our study affirmed that low concentration of genistein (1mg/kg BW) was the effective doses in female reproductive function by increasing antioxidant activity and thereby decreasing ROS production.

ROS have important roles in modulating many reproductive physiological functions including folliculogenesis, oocyte maturation, ovulation, fertilization, implantation, ovarian steroidogenesis, progesterone production, and luteolysis (7, 22). This function improved by making a suitable balance between oxidant and antioxidant mechanism. In contrast, the overproduction of ROS or oxidative stress interferes with many pathological conditions of reproduction such as infertility, abortion, preeclampsia, endometriosis, and PCOS (23).

In a previous meta-analysis, the MDA level, a direct oxidant marker, was ∼47% higher in PCOS women than in controls, suggesting that MDA level might be one of best markers to represent the oxidative stress status in PCOS models (24).

Our results in accordance with Pandey and colleagues indicated that the MDA levels as a marker of lipid peroxidation increased in within the plasma and ovaries of PCOS rats, while antioxidant activities (TAC, GPx, and SOD markers) decreased in contrasts to Daneasa and colleagues results (22).

It is seen that oxidant–antioxidant balance was disrupted in the PCOS rats and thereby could damage biologic molecules and cell membrane and resulted in increasing the MDA level and thereby failed the antioxidants activities.

In addition, the extension of oxidative stress production or inadequate removal of ROS resulted in Ca2+ influx into cells that induced further cell injury and increased DNA strand breakage (25, 26). Therefore, the histological examinations affirmed that both evaluation and protection of oxidative stress are very important in PCOS models.

Antioxidants supplementation as a therapeutic method implement critical role in three approaches: minimize or prevent, repair and remove oxidative damage (27). Antioxidants are categorized as enzymatic and non-enzymatic. The enzymatic antioxidants include SOD, GPx, and CAT (23). SOD is the important oxidant scavengers with antioxidant effect against superoxide anion that reduces the superoxide anion radicals and catalyzes convert them to H2O2 (28). GPx plays an effective role in the peroxyl scavenging mechanism that converts H2O2 (produced by SOD) into water (23, 28).

Genistein is a phytoestrogen that is similar to the chemical structure of 17β-estradiol. It has been known as a powerful antioxidant and oxidant scavenger that inhibits oxygen radicals and hydrogen peroxide production through the activation of SOD and GPx enzymes in several organs (12).

The elevation of antioxidant activity strongly suggests that ovarian tissue was protected against oxidative damages in PCOS conditions. Therefore, antioxidants supplementation in PCOS condition positively contributes to the management of PCOS and its complications.

However, Zhuang *et al*. showed four-weeks treatment with genistein improving the development and viability of rat ovarian follicles (29). Genistein may exert its protective effects through many biochemical and multidirectional pathways. Genistein may decrease the pool of ROS and act as free radical scavengers in the ovary due to their polyphenolic structures and hydroxyl group (28). Moreover, genistein exerts antioxidant effects through the modulation of transcription factors such as nuclear respiratory factor (Nrf), nuclear factor kappa-light-chain-enhancer of activated B cells (NF-kB) (30, 31), and anti-inflammatory effects (32).

The previous studies indicated that inappropriate levels of gonadotropins and sex hormones induced polycystic ovaries (31, 33). On the other hand, follicle-stimulating hormone (FSH) levels decreased and Luteinizing hormone (LH) levels increased in PCOS patients. The imbalances levels of FSH and LH impair follicular growth and lead to anovulation in PCOS (31). Moreover, high levels of LH lead to excessive secretion of estrogen and testosterone by suppressing aromatase activity (conversion of androgen hormones into estrogens) and thereby resulting to the disruption of sexual cycle and ovulation in PCOS (33, 34).

Kafali and colleagues demonstrated that the inappropriate secretion of FSH and LH is due to an inadequate interaction between granulosa and theca cells in PCOS ovaries (35). The damaged granulosa and theca cells in our histological observation affirmed the abnormal secretion of gonadotropins in PCOS rats.

The protective effects of genistein may be mediated not only through antioxidant activity but also by retaining follicular steroidogenesis and ovarian cycle, which is disrupted by PCOS conditions (36). Genistein is a natural inhibitor of ovarian estrogenic activity by binding to the estrogen receptor (ER) with the polyphenolic structure (11, 28) and decrease testosterone by stimulation of aromatase activity (34).

In addition, genistein may affect the hypothalamic pituitary axis and increase the gonadotrophin-releasing hormone (GnRH) production and lead to reducing the secretion of gonadotropins (3). Moreover, the level of FSH increased and affect the follicular growth and subsequently stimulated granulosa cells for the production of estrogen and preserve follicular reserve (30).

## 6. Conclusion

Our study revealed that there is an increased oxidative stress and decreased antioxidant activity in rat PCOS model, and suggests that oxidative stress as an important pathological feature of PCOS could induce formation of cystic follicles and disrupt follicular structure. Thus, these physical, biochemical and histological results clearly demonstrated that treatment with genistein promotes follicular growth by achieving an adequate antioxidant defense system and scavenging free radicals, thereby reducing the harmful effects of excess of oxidative stress on follicular structure in the PCOS model. Further researches are recommended to clarify the effects of genistein on oxidative stress in the PCOS model.

##  Conflict of Interest

The authors have no conflicts of interest to declare.
